# *Sambucus nigra* extracts inhibit infectious bronchitis virus at an early point during replication

**DOI:** 10.1186/1746-6148-10-24

**Published:** 2014-01-16

**Authors:** Christie Chen, David M Zuckerman, Susanna Brantley, Michka Sharpe, Kevin Childress, Egbert Hoiczyk, Amanda R Pendleton

**Affiliations:** 1 Division of Natural Science and Mathematics, Oxford College of Emory University, Oxford, GA 30054, USA; 2 W. Harry Feinstone Department of Molecular Microbiology and Immunology, The Johns Hopkins University School of Public Health, Baltimore, MD 21205, USA; 3 Department of Mathematics, Sciences & Engineering, Amarillo College, Amarillo, TX 79178, USA

**Keywords:** Infectious bronchitis virus, Coronavirus, *Sambucus nigra*, *Nigella sativa*, *Rhodiola rosea*

## Abstract

**Background:**

Infectious bronchitis virus (IBV) is a pathogenic chicken coronavirus. Currently, vaccination against IBV is only partially protective; therefore, better preventions and treatments are needed. Plants produce antimicrobial secondary compounds, which may be a source for novel anti-viral drugs. Non-cytotoxic, crude ethanol extracts of *Rhodiola rosea* roots, *Nigella sativa* seeds, and *Sambucus nigra* fruit were tested for anti-IBV activity, since these safe, widely used plant tissues contain polyphenol derivatives that inhibit other viruses.

**Results:**

Dose–response cytotoxicity curves on Vero cells using trypan blue staining determined the highest non-cytotoxic concentrations of each plant extract. To screen for IBV inhibition, cells and virus were pretreated with extracts, followed by infection in the presence of extract. Viral cytopathic effect was assessed visually following an additional 24 h incubation with extract. Cells and supernatants were harvested separately and virus titers were quantified by plaque assay. Variations of this screening protocol determined the effects of a number of shortened *S. nigra* extract treatments. Finally, *S. nigra* extract-treated virions were visualized by transmission electron microscopy with negative staining.

Virus titers from infected cells treated with *R. rosea* and *N. sativa* extracts were not substantially different from infected cells treated with solvent alone. However, treatment with *S. nigra* extracts reduced virus titers by four orders of magnitude at a multiplicity of infection (MOI) of 1 in a dose-responsive manner. Infection at a low MOI reduced viral titers by six orders of magnitude and pretreatment of virus was necessary, but not sufficient, for full virus inhibition. Electron microscopy of virions treated with *S. nigra* extract showed compromised envelopes and the presence of membrane vesicles, which suggested a mechanism of action.

**Conclusions:**

These results demonstrate that *S. nigra* extract can inhibit IBV at an early point in infection, probably by rendering the virus non-infectious. They also suggest that future studies using *S. nigra* extract to treat or prevent IBV or other coronaviruses are warranted.

## Background

Avian infectious bronchitis virus (IBV), a gamma-coronavirus, infects the respiratory tract of chickens and causes the production of eggs with deformed and weakened shells [1,2]. The poultry and egg industries have consequently suffered large economic losses due to IBV infections [3,4]. Current vaccination strategies target specific serotypes of the virus. However, vaccines have not proven wholly effective in protecting against new infections due to the highly recombinant nature of the virus [5,6]. More efficient methods of IBV prevention or treatment are clearly needed. Plant extracts may be a potential source of agents for defending against IBV.

Historically, plant extracts have been widely used to treat various medical conditions [7-9]. Some of the best-known examples include quinine isolated from *Cinchona pubescens* (Cinchona tree) for treating malaria, digoxin from *Digitalis purpurea* (foxglove) for treating cardiac conditions, morphine from *Papaver somniferum* (opium poppy) used for pain, and aspirin synthesized from the bark of various *Salix* (willow) species. In many of these cases, the active chemicals isolated from these plants have been the basis for developing additional medications that are used today. Additionally, myriad plant extracts have shown activity, both *in vitro* and *in vivo*, against a large range of viral pathogens, including hepatitis B and C viruses, herpes simplex virus, influenza virus, poliovirus, dengue viruses, and human immunodeficiency virus [10]. Plant secondary metabolites, particularly polyphenols, are also increasingly recognized as potent antimicrobials [11]. In some cases this ability to use plant metabolites to combat animal pathogens may rise from the similarities in plant and animal innate immune systems [12]. Some commonalities include the use of similar pathogen recognition receptors and MAP-kinase signaling pathways to upregulate cellular immune responses, as well as reactive oxygen species and defensins to protect against invading microbes. Therefore, it is not surprising that the secondary metabolites used by plants for their own defense have been effective inhibitors, in some cases, of animal infectious agents [13]. One such secondary metabolite is catechin. In *Picea abies* (Norway spruce) and *Carmellia sinensis* (Chinese tea leaf), catechin-synthesizing genes are upregulated in response to fungal infection and are correlated with increased resistance to infection [14,15]. In humans, ingestion of or gargling with catechin-containing plant extracts results in lower rates of influenza virus infection [16,17]. Quercetin is another secondary metabolite involved in plant and animal pathogen defense. Treatment with quercetin reduces susceptibility of *Arabidopsis thaliana* (mouse-ear cress) to *Pseudomonas syringae* infection [18]. *In vitro* and *in vivo* studies have both shown that quercetin and its derivatives inhibit influenza virus and poliovirus replication, while *in vitro* treatment of the human pathogen, *Salmonella enterica*, results in microbe death [19-24].

The use of plant extracts as an alternative or supplementary IBV treatment or prevention strategy has not been extensively investigated. The range of plants that have been surveyed for their potential as anti-IBV agents is also limited, although, purified compounds isolated from *Glycyrrhiza radix* (licorice root) [25] and *Forsythia suspensa* (weeping forsythia) [26] have shown effectiveness against IBV *in vitro*. However, the use of these extracts or the active ingredients from these extracts for long-term treatment or prevention strategies poses some toxicity concerns [27-29]. These concerns, combined with the difficulties often encountered when translating *in vitro* research into *in vivo* treatments [30], suggest that *in vitro* identification of a number of different antiviral plants for future *in vivo* studies is important.

This study investigated the effects of extracts of three plant species – *Rhodiola rosea* (goldenroot), *Nigella sativa* (black cumin) and *Sambucus nigra* (common elderberry) – on avian IBV replication. To our knowledge, our study is the first to test the effects of these plants on IBV replication. We chose to study these plants due to their known antiviral properties. For example, *R. rosea* extract has shown antiviral activity against coxsackievirus B3 by preventing the virus from attaching and entering host cells [31]. *R. rosea* extracts also contain a number of antiviral chemicals, including gallic acid, caffeic acid, chlorogenic acid, and catechin [32], which have inhibited the replication of human rhinoviruses [33], hepatitis B virus [34], and influenza virus [16,17]. *N. sativa* extract has shown antimicrobial properties against *Escherichia coli*, *Bacillus subtilis*, and other bacteria [35]. Studies of murine cytomegalovirus infection and hepatitis C infection lend support to the plant’s antiviral potential *in vivo*, as well [36,37]. Additionally, *N. sativa* compound extracts*,* especially its saponins, alkaloids, and flavonols, show similarities with known antiviral chemicals [38-40]. Finally, *S. nigra* extract has successfully inhibited influenza A and B viruses *in vitro* and *in vivo*[41]. *S. nigra* extracts are also characterized by a high content of antiviral flavonoid anthocyanins [42]. Additionally, the antiviral compound quercetin is largely present in both *S. nigra* and in *Amelanchier alnifolia* (Saskatoon serviceberry) [43], a known inhibitor of the bovine coronavirus, *in vitro*[44]. Combined, these studies suggested that extracts of *R. rosea*, *N. sativa*, and *S. nigra* might possess broad antimicrobial or antiviral properties.

Here we show that non-cytotoxic, crude ethanol extracts of *R. rosea* roots and *N. sativa* seeds did not inhibit IBV infection *in vitro*, while *S. nigra* fruit extracts inhibited IBV by several orders of magnitude. This inhibition was dose-responsive in that it decreased with decreasing *S. nigra* extract concentrations and increased with decreasing virus concentrations. Treatment of virus with *S. nigra* extracts prior to infection was necessary, but not sufficient, for full virus inhibition. Additionally, electron microscopy of virions treated with *S. nigra* extracts showed compromised envelopes and the presence of membrane vesicles. These results demonstrate that *S. nigra* extract can inhibit IBV at an early point in infection and suggest that it does so by compromising virion structure. Overall these studies identified a plant extract with previously unknown effects against IBV, which could potentially lead to effective treatments or prevention of this or similar coronaviruses.

## Methods

### Cells and viruses

Vero cells were maintained in high-glucose Dulbecco’s modified Eagle’s medium (DMEM) (Invitrogen Corporation, Carlsbad, CA) supplemented with 10% fetal calf serum (Atlanta Biologicals, Norcross, GA) and 0.1 mg/ml Normocin (Invivogen, San Diego, CA). The previously described Vero-adapted Beaudette strain of IBV [45] was used in all IBV infection experiments. Infections and titers were performed with Vero cells.

### Preparation of plant extracts

0.6 g of *R. rosea* powdered root (Starwest Botanicals Sacramento, CA) was incubated in 5 ml of 70% ethanol (Sigma-Aldrich, St. Louis, MO) for 24 h at room temperature [32]. 1.5 g of *N. sativa* seeds (Frontier Natural Products Co-op, Norway, IA) was homogenized in 10 ml of 85% ethanol and incubated for 7 d at room temperature [46,47]. 32.0 g of *S. nigra* fruit (San Francisco Herb Company, San Francisco, CA) was homogenized in 40 ml of 80% ethanol and incubated for 4 d at room temperature [48]. Following these incubations, extract solutions were centrifuged at 1900 × *g* for 5 min at room temperature to remove debris and the remaining supernatant was syringe filtered through a 0.22 μm polyvinylidene fluoride membrane (Fisher Scientific Company, Fair Lawn, NJ). All extract solutions were stored at 4°C.

### Cytotoxicity assays

Cells were plated in 35-mm dishes in duplicate for approximately 1 d before being treated with plant extracts for 48 h. Concentrations ranged from 7.5 × 10^-5^ g/ml to 1.2 × 10^-3^ g/ml for *R. rosea* extract, from 9.4 × 10^-5^ g/ml to 1.5 × 10^-3^ g/ml for *N. sativa* extract, and from 5.0 × 10^-4^ g/ml to 8.0 × 10^-3^ g/ml for *S. nigra* extract. The final concentration of solvent was kept constant in all wells at 0.04% ethanol for *R. rosea* extract treatments, 0.2% ethanol for *N. sativa* extract treatments, and 0.4% ethanol for *S. nigra* extract treatments. At 48 h post-treatment, supernatants containing dead cells were collected and combined with adherent cells that had been harvested using 0.05% trypsin (Invitrogen Corporation) in Dulbecco’s phosphate-buffered saline (Sigma-Aldrich, St. Louis, MO). 20 ml of this solution was then combined with an equal volume of 0.6% trypan blue (Sigma-Aldrich). The number of live cells per ml in each dish was counted in duplicate using light microscopy and a hemocytometer. The relative cell viability was calculated as live cells per ml in extract-treated dishes relative to solvent-treated dishes.

### Infection in the presence of plant extracts

To screen for anti-IBV effects, cells were plated in 35-mm dishes for approximately 2 d before being treated with 3.75 × 10^-4^ g/ml of *N. sativa* extract, 1.5 × 10^-4^ g/ml of *R. rosea* extract, or 4.0 × 10^-3^ g/ml of *S. nigra* extract for 24 h. Control cells for *R. rosea*, *N. sativa*, and *S. nigra* extract treatments were incubated in final concentrations of 0.04% ethanol, 0.2% ethanol and 0.4% ethanol, respectively. Prior to infection with IBV, virus was incubated with these same concentrations of plant extract (or solvent alone) for 20 min at room temperature. IBV infection was then performed at a multiplicity of infection (MOI) of either 1 or 0.1 by allowing virus to absorb to cells in a small volume of serum-free DMEM supplemented with plant extract or solvent alone for 1 h at 37°C. Cells were then transferred to fresh DMEM supplemented with 10% fetal calf serum, antibiotics, and plant extract or solvent for an additional 24 h. Viral cytopathic effect was then assessed visually using light microscopy, and virus-infected supernatants and cells were harvested separately. Supernatants were collected, centrifuged at 1900 × *g* for 5 min at room temperature to remove cellular debris, and stored at -80°C until virus titers could be determined. Cells were transferred to fresh DMEM supplemented with 10% fetal calf serum and antibiotics before being lysed by three rounds of freeze-thaw. Centrifugation at 1900 × *g* for 5 min at room temperature removed cellular debris and the remaining supernatant was stored at -80°C until virus titers could be determined.

After initial screening, the following *S. nigra* extract treatments were assessed for their ability to inhibit IBV either alone or in combination: (1) exposing cells to extract prior to infection, (2) exposing cells to extract following infection, (3) exposing virus to extract prior to infection, (4) exposing both cells and virus to extract during infection. Infections were done at an MOI of 0.1 as indicated above, except that exposure to solvent alone was substituted for exposure to *S. nigra* extract if a specific treatment was omitted. For example, to determine the effects of only exposing cells to *S. nigra* extract prior to infection, cells were first incubated with 4 mg/ml of *S. nigra* extract for 24 h prior to infection. Virus was then incubated in solvent alone for 20 min prior to infection and solvent was present during infection. Cells were then incubated in solvent alone for an additional 24 h following infection before being harvested, as described above.

### Plaque assays

Virus titers were quantified *via* plaque assay. First, serial dilutions of virus were absorbed to confluent Vero cells for 1 h in a small amount of serum free DMEM. Virus was then removed from cells and an agarose overlay (equal volumes of 2× DMEM and 1.8% melted agarose (Invitrogen Corporation)) was added. After 2 d, an additional agarose overlay containing 0.015% neutral red (MP Biomedicals, LLC, Solon, OH) was added to cells. Approximately 24 h later, clear plaques were counted and virus titers were calculated in particle forming units/ml (pfu/ml).

### Electron microscopy

To purify virus, 30 ml of cell culture supernatant was overlaid on 4 ml of 20% sucrose in TNE buffer (50 mM Tris, pH 7.4, 100 mM NaCl, 1 mM EDTA) and 2 ml of 55% sucrose in TNE in an SW 28 tube (Beckman-Coulter, Brea, CA). Samples were spun for 3 h at 25 k RPM in an SW 28 rotor. Purified virus was collected from the 20%-55% sucrose interface, diluted with TNE and pelleted for 2 h at 55 k RPM in an SW 55Ti rotor. Pellets were resuspended in 40–60 μl TNE and kept on ice for immediate use.

Purified virus was treated with 8.0 × 10^-3^ g/ml of *S. nigra* extract or 0.8% ethanol as a vehicle control in PBS for 15 min at room temperature. Samples were then spotted onto a glow discharged, carbon coated copper grid (Electron Microscopy Sciences, Hatfield, PA) and incubated for 2 min. Grids were rinsed with water, and stained for 1 min with 2% phosphotungstinic acid, pH 7.4. Samples were examined on a Hitachi 7600 transmission electron microscope under 80 kV, and micrographs collected using AMT Image Capture Engine software controlling an AMT ER50 5 megapixel CCD camera (Advanced Microscopy Techniques Corp., Danvers, MA).

### Ethical approval

The research protocol used for this study was approved by the Health & Biosafety Committee at Emory University (Biosafety File #: 08-2528-11). No human or animal subjects were used.

## Results

### Determining non-cytotoxic concentrations of plant extracts

**Figure 1:**
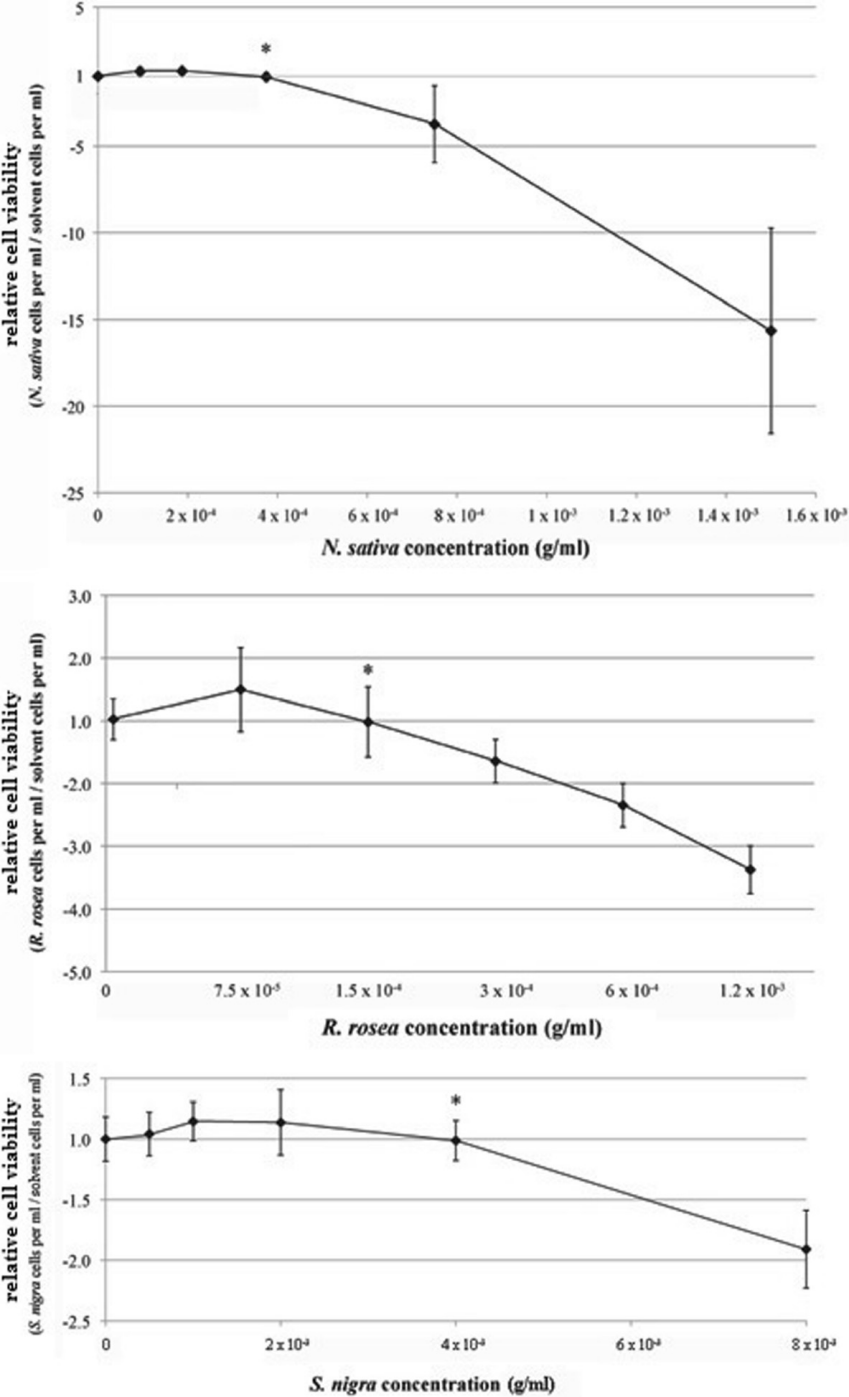
**Determining non-cytotoxic concentrations of each plant extract.** Vero cells were treated for 48 hours with the indicated concentration of plant extract. Independent cytotoxicity assays were performed three times, with four replicates per assay, using trypan blue staining. Error bars represent standard deviation. Starred data points represent the highest concentration of extract that was not significantly different from the control by a student’s t-test (p > 0.05). These starred concentrations were used in all subsequent infection assays, unless noted otherwise.

Screening of plant extracts for antiviral potential must be done using non-cytotoxic concentrations of extract. Therefore, cytotoxicity assays with trypan blue staining were performed. Cells were treated for 48 h with the indicated concentration of *N. sativa*, *R. rosea*, or *S. nigra* extracts and the number of live cells for each concentration of extract, relative to solvent treatment alone, was determined. For all plant extracts, the number of live cells decreased with increasing concentrations of extract in a dose-responsive manner (Figure 1). The highest concentration of plant extract that did not significantly decrease the number of live cells, relative to controls, was used for all subsequent antiviral screening.

### *N. sativa* and *R. rosea* extracts do not inhibit IBV, while *S. nigra* extracts do

Antiviral agents may exhibit an effect *via* myriad mechanisms. Therefore, screening was performed using extract before, during, and after infection to maximize the possibility of detecting antiviral action. Cells were treated for 24 h prior to infection with the indicated concentration of extract. Virus was treated for 20 min prior to infection and extract was present during the 1 h absorption of virus to cells. Cells were then treated for an additional 24 h post-infection (p.i.). Treatment with solvent alone was used as a control. At 24 h p.i. cells were visually assessed for viral cytopathic effect (CPE). Supernatants and cells were harvested separately and viral titers were quantified.

**Figure 2:**
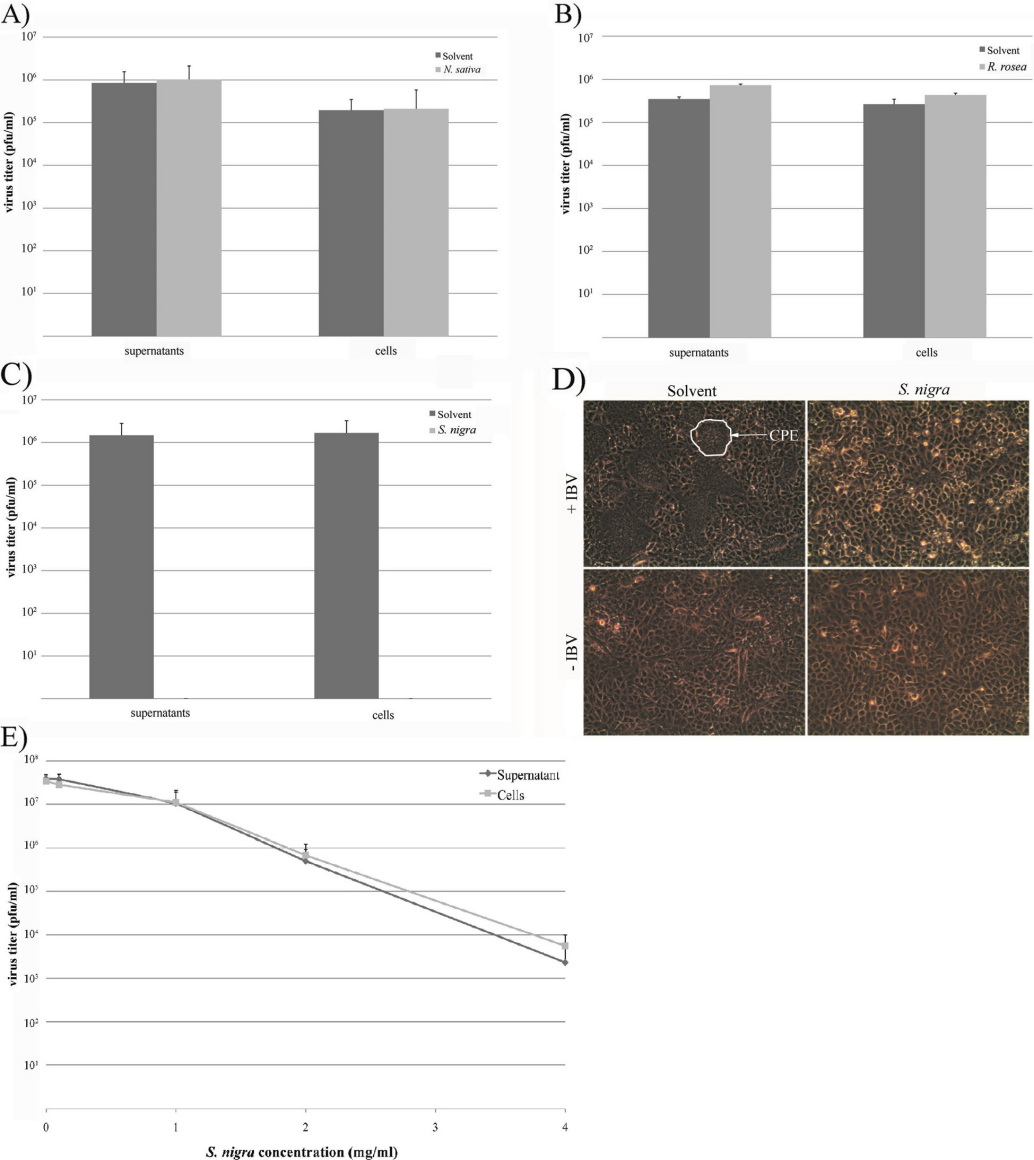
***N. sativa *and *R. rosea *extracts do not inhibit IBV, while *S. nigra *extracts do. A – D)** Cells were pretreated for 24 h and virus for 20 min with 3.75 × 10^-4^ g/ml *N. sativa* extract, 1.5 × 10^-4^ g/ml *R. rosea* extract, 4.0 x 10^-3^ g/ml *S. nigra* extract, or solvent alone prior to infection in the presence of extract. The same concentration of extract was also present during virus absorption to cells. Cells were then treated for an additional 24 h p.i. with the same concentration of extract. Independent infections with IBV were performed three times at an MOI of 0.1, with two replicates per assay. **E)** Cells were treated as for **A–D**, except that different concentrations of *S. nigra* extract were used, as indicated. Additionally, IBV infections were performed at an MOI of 1. **A**, **B**, **C**, **E**) Quantitation of virus titers at 24 h p.i. was done by plaquing in duplicate using neutral red staining. **D)** Visualization of viral CPE was done at 24 h p.i. *via* light microscopy. **A) ***N. sativa*, **B) ***R. rosea, ***C – E) ***S. nigra.*

Virus titers of the *N. sativa* extract-treated supernatants and cells were not significantly different from controls (Figure 2A). Unexpectedly, *R. rosea* extract-treated supernatants and cells showed a small, yet reproducible, two-fold increase in virus titers (Figure 2B). On the other hand, *S. nigra* extract-treated cells showed no detectable CPE at an MOI of 0.1 and a reduction of virus titers by six orders of magnitude (Figures 2C & 2D). Inhibition was not as great in *S. nigra* extract-treated samples when a higher MOI of 1 was used (Figure 2E). However, this inhibition was still large, reducing viral titers by approximately four orders of magnitude, relative to solvent-treated samples. Virus titers also decreased with increasing *S. nigra* extract concentrations in a dose-responsive manner, indicating that *S. nigra* extract treatment was responsible for virus inhibition.

### *S. nigra* extracts inhibit IBV at an early step in the infection process

**Figure 3:**
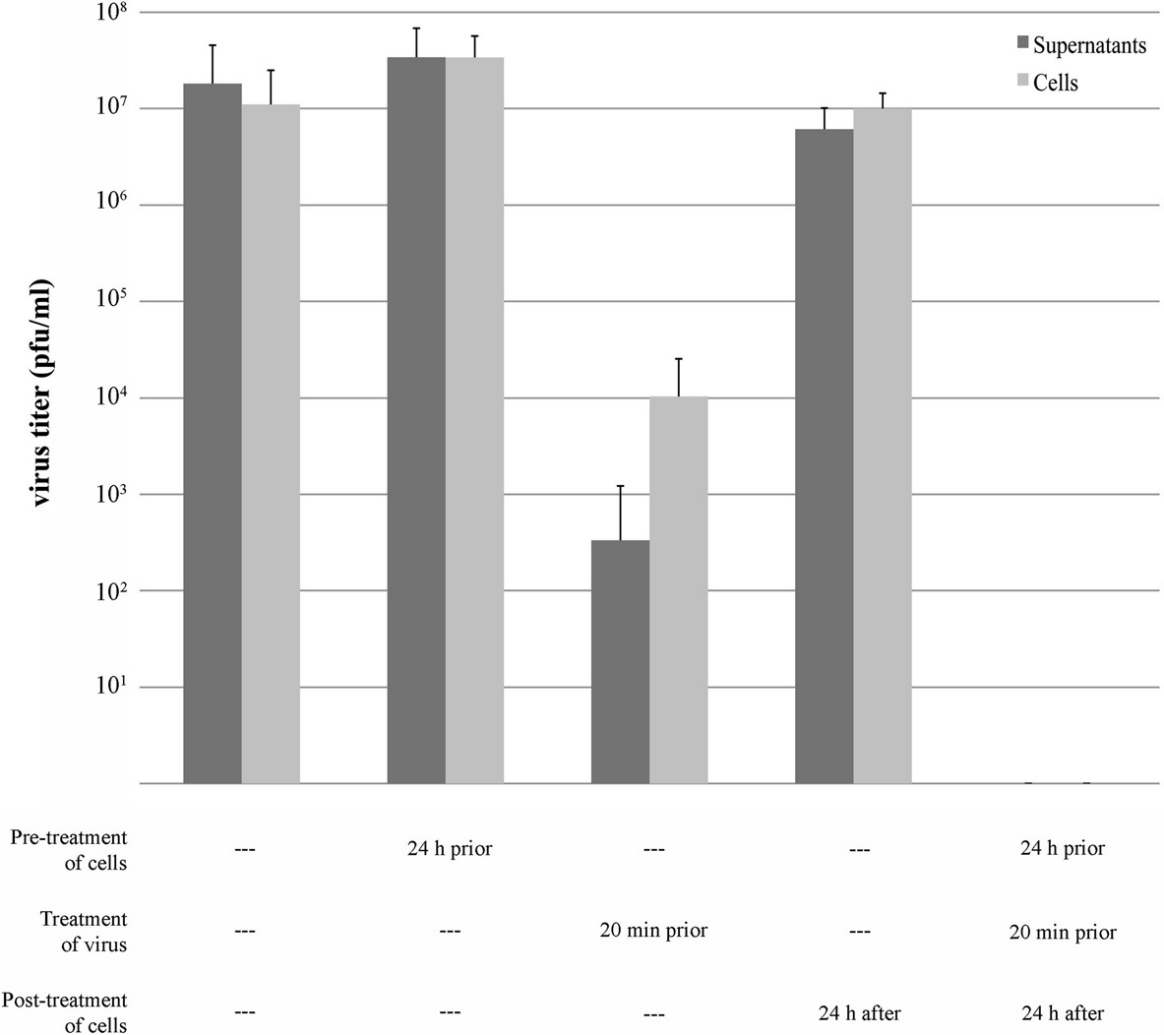
**Pre-treatment of IBV with *S. nigra *extracts dramatically reduces viral titers.** Cells and virus were treated with 4.0 x 10^-3^ g/ml of *S. nigra* extract as indicated below. Infection was done at an MOI of 0.1. Quantitation of virus titers at 24 h p.i. was done by plaquing in duplicate using neutral red staining. Independent infections with IBV were performed three times, with two replicates per assay.

To begin uncovering the mechanism by which *S. nigra* extracts inhibited IBV, we assessed the impact of shortened *S. nigra* extract treatments on IBV replication. A series of infections were done in which only cells were treated with extract prior to infection (pre-C), only virus was treated prior to infection (pre-V), or only treatment following infection was done (post). The pre-C treatment did not result in any reduction in virus titer relative to treatment with solvent alone (Figure 3). Similarly, virus titers were not reduced in the cells of samples that received only the post treatment. However, the post treatment did result in a modest, three-fold reduction in titers of the supernatants. On the other hand, the pre-V treatment resulted in a titer reduction of over three orders of magnitude in the cells and over four orders of magnitude in the supernatants. Clearly, out of the three shortened treatments tested, the pre-V treatment alone showed the greatest inhibition. However, this treatment was not sufficient for reducing virus titers to the same level as when all three treatments were combined.

**Figure 4:**
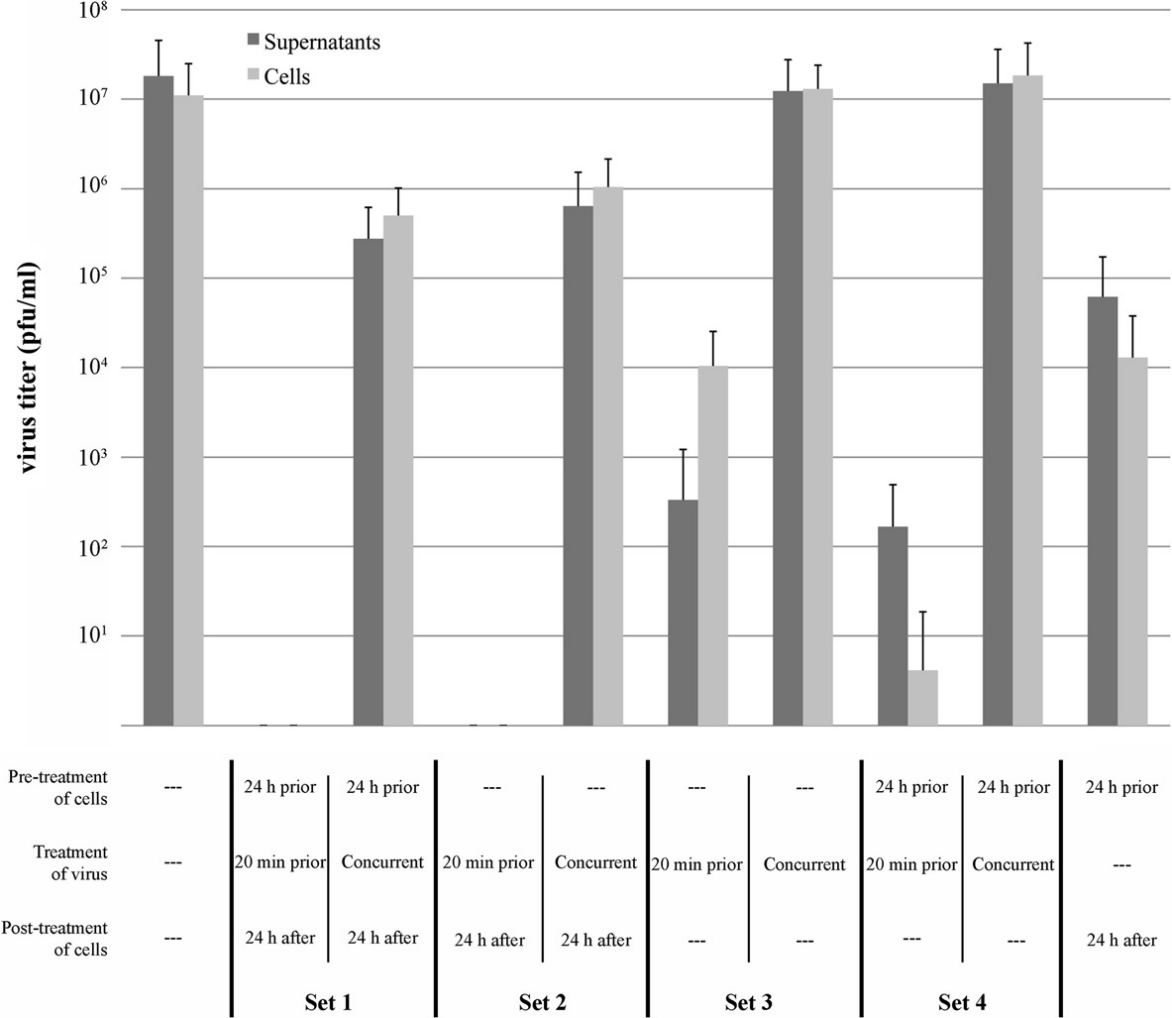
**Treating IBV with *S. nigra *extracts prior to infection is necessary for full virus inhibition and works synergistically with treating cells after infection.** Cells and virus were treated with 4.0 x 10^-3^ g/ml of *S. nigra* extract as indicated below. Infection was done at an MOI of 0.1. Quantitation of virus titers at 24 h p.i. was done by plaquing in duplicate using neutral red staining. Independent infections with IBV were performed three times, with two replicates per assay.

To further explore the effects and potential synergy of different timings of extract exposure, another series of infections was done with varying extract treatment scenarios, as indicated (Figure 4). Results from these experiments revealed that combining pre-V treatment with post treatment worked together to fully inhibit IBV replication. The pre-C treatment was not necessary for full virus inhibition, nor did it impact the viral titer of the supernatant. However, it did work synergistically with pre-V treatment to reduce viral titers in the cells an additional three orders of magnitude, as compared to pre-V treatment alone. In addition, exposing virus to *S. nigra* extract at the time of infection did not reduce virus titers, unless it was combined with the post treatment. In every combination of treatments, pre-treating the virus with *S. nigra* extract greatly increased virus inhibition. Finally, combining the pre-C and post treatments did result in a further two orders of magnitude titer reduction in the supernatants and cells, when compared to post treatment alone. Taken together these results indicate that some treatments worked together to fully inhibit IBV replication. Importantly, the necessity and large effect seen with pre-V treatment indicated that one mechanism of inhibition occurs at an early step of the IBV replication cycle.

### *S. nigra* extract compromises IBV virion structure

**Figure 5:**
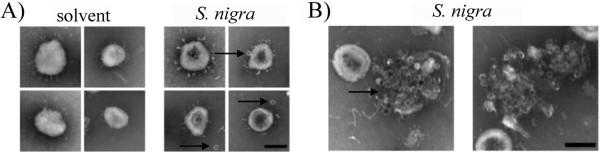
**Treating IBV with *S. nigra *extracts compromises virion structure.** Virus was treated with 8.0 x 10^-3^ g/ml of *S. nigra* extract or solvent (vehicle) alone for 10 min before being prepared for transmission electron microscopy with negative staining. **A)** Most frequent virion structures observed. bar = 100 nm. Arrows indicate vesicle structures. **B)** Aggregates of vesicle structures observed only in virus samples treated with *S. nigra*. bar = 100 nm.

To explore if the extracellular effect of *S. nigra* extract on IBV infectivity was due to physical disruption of the virion, virus samples treated with *S. nigra* extract or solvent alone were negative stained and examined by transmission electron microscopy. Intact virions with uncompromised envelopes and characteristic spike protein profiles were easily identified in solvent treated samples (Figure 5A). By contrast, treatment of the virus with *S. nigra* extract resulted exclusively in virions with damaged envelopes. The profiles of the spike proteins appeared unaffected in the treated samples, but the membranous envelope appeared to have been compromised (Figure 5A). Additionally, in the extract treated samples, many spheres, resembling membrane vesicles, were seen surrounding the virions (Figure 5A) or clustered together in large aggregates (Figure 5B). These vesicles were of relatively uniform size (24.4 +/- 1.7 nm, n = 58) and were only apparent in extract treated virions, and not in solvent treated virus, or extract alone (Figure 5 and data not shown). Taken together, these data indicate that the pre-treatment of IBV with *S. nigra* extract results in extensive membrane damage to the virus, likely rendering it non-infectious.

## Discussion

Vaccination against IBV, a pathogen that causes large economic losses among the egg and poultry industries, has not proven wholly effective; therefore, alternative treatment or prevention strategies are needed. Here we screened non-cytotoxic (Figure 1), crude ethanol extracts from *S. nigra* berries, *N. sativa* seeds, and *R. rosea* roots for antiviral effects. Only *S. nigra* extracts inhibited viral replication, reducing viral titers by four to six orders of magnitude in a dose-dependent manner (Figure 2). *S. nigra* extract treatment of only virus prior to infection drastically inhibited the virus (Figure 3), indicating that *S. nigra* extract inhibits IBV at an early point in the infection process. Electron microscopy of *S. nigra* extract-treated IBV revealed compromised virion structures and membranous vesicles (Figure 5), which were not present in the extract alone. Therefore, *S. nigra* extract disrupts IBV virion structure, likely rendering it non-infectious.

Our results raise questions about which compounds within the crude *S. nigra* extract inhibit IBV, as well as their mechanisms of action. Polyphenols are a likely source of this inhibition, as plants with high polyphenol concentrations often have antiviral properties [11]. In fact, two flavonols extracted from *S. nigra* berries can bind to virions from specific influenza virus strains and prevent infection *in vitro*[48], although whether these flavonols disrupted virion structure is unknown. Perhaps these or similar compounds in our *S. nigra* extract also inhibited IBV. Intriguingly, *S. nigra* extract has now been shown to inactivate two enveloped viruses, in the case of IBV by compromising its membrane directly. The membranes of these two viruses are chemically distinct, with IBV membranes being derived from the endoplasmic reticulum Golgi intermediate compartment, while influenza membranes are derived from the plasma membrane. These results suggest that *S. nigra* extract may have broad anti-viral effects against other enveloped viruses.

In addition to polyphenols, lectins are commonly found in plant extracts and often show antiviral activity by binding to viral proteins or host receptors, preventing their interaction [49-54]. *S. nigra* berry extracts are known to contain three plant lectins [55-59]. Two of these lectins possess specificity for galactose and N-acetylgalactosamine, while the other one preferentially binds α2,6-linked sialic acid. Although IBV, a gamma-coronavirus, depends upon sialylated host receptors for entry into cells, it specifically uses α2,3-linked moieties, not α2,6-linked moieties [60]. Therefore, it is unlikely that *S.nigra* lectins block access to host-cell receptors used by IBV. Our results support this idea, since treatment of cells prior to infection had no effect on viral replication (Figure 3). On the other hand, IBV proteins, such as the spike protein, contain several consensus sequences that signal the addition of N-linked oligosaccharides [61]. Possibly, *S. nigra* lectins could bind directly to viral proteins and inhibit infection. Lectins bound to the virions of both an alpha- and beta-coronavirus did inhibit infection [62], lending support to this idea. How binding by *S. nigra* lectins and virion disruption (Figure 5) would be related is unclear and might occur by separate mechanisms.

While *N. sativa* and *R. rosea* extracts did not inhibit IBV, many of their phytochemicals are thought to be antiviral. For example, *N. sativa* seed extracts predominantly contain saponins, glycosides, terpenoids and alkaloids [38,63-67], many of which are similar to known antiviral chemicals [38-40,68]. On the other hand, *R. rosea* root extracts consist of many kaempferol, herbacetin, dihydromyricetin, and myricetin derivatives [32]. Of these *R. rosea* compounds, kaempferol, gossypetin, and salidroside have shown strong antiviral effects against influenza and Coxsackie viruses [69,70]. *S. nigra* is also rich in cyanidin, kaempferol, myricetin, dihydromyricetin, and quercetin derivatives [42,71,72], making it much more similar chemically to *R. rosea* than to *N. sativa*. However, chemicals that are found in *S. nigra* berry extracts, but not in either *R. rosea* or *N. sativa* extracts, are particularly attractive candidates for future tests into the chemical nature of *S. nigra* extract inhibition. These *S. nigra* chemicals include several cyanidin derivatives; 3-, 4-, and 5-caffeoylquinic acid; kaempferol 3-rutin; rutin; pelargonidin 3-glucoside; isorhamnetin 3-rutin; and isorhamnetin 3-glucoside. Cyanidins, kaempferols, and isorhamnetins are known antiviral chemicals [68]. Additionally, the two flavonols (5,7,3’,4’-tetra-O-methylquercetin and 5,7-dihydroxy-4-oxo-2-(3,4,5-trihydroxyphenyl)chroman-3-yl-3,4,5-trihydroxycyclohexanecarboxylate), which bind to and inhibit influenza virus [48], are found in *S. nigra* and not in *R. rosea* or *N. sativa*, making them potential candidates as well. Alternatively, testing different fractions of *S. nigra* extracts for antiviral capabilities, along with direct chemical identification, could identify which, if any, of these chemicals are responsible for the early inhibition of IBV replication. In addition, other plant extracts with chemicals that are similar to those in *S. nigra* extracts might also be considered for future anti-IBV tests. For example, extracts from *A. alnifolia* berries, branches, and leaves have chemicals (3-carreolyquinic acid and cyanidin 3-glucoside) that are found in *S. nigra* but not in *R. rosea* or *N. sativa*[42,71,73]. And indeed, *A. alnifolia* branch extracts inhibited the bovine coronavirus *in vitro*[44]. Finally, a currently unidentified chemical or combination of chemicals may be responsible for the ability of *S. nigra* extract to compromise IBV virion structure. One possibility may be cholesterol chelators, since they are known to compromise the membrane integrity of other viruses, resulting in a loss of infectivity [74]. Currently, none of the chemicals known to be present in *S. nigra* berry extracts chelate cholesterol or have vesiculating effects on lipid membranes; however, future studies may demonstrate otherwise.

Various combinations of *S. nigra* extract treatments also showed synergistic inhibition. For example, complete inhibition occurred when pre-treatment of virus was done in combination with post-infection treatment (Figure 4). Potentially, this synergy is due solely to compromised virion structure, since these experiments were done at a low MOI and allowed more than one round of replication to occur. Specifically, virions that survive the pre-treatment intact would be competent for infection, and their progeny would face no further challenge from the extract in the absence of post-infection treatment. Alternatively, the synergistic inhibition of infected cells seen when pre-treatment of virus and pre-treatment of cells were combined may indicate that more than one mechanism is at work and that more than one active compound is present in the crude extract. Again, testing of *S. nigra* extract fractions will help explore this possibility.

If polyphenols in *S. nigra* extract are the cause of inhibition, growing conditions and cultivars could greatly affect the antiviral properties of the plant extracts. For example, the Korsør, Haschberg, and Rubini cultivars of *S. nigra* vary in their phenolic concentrations [42,71,75]. In addition, within each cultivar of *S. nigra*, the polyphenols vary throughout different growing seasons [71]. If *in vivo* tests also demonstrate IBV inhibition by *S. nigra* extract, identifying the most efficient cultivar and growing conditions for *S. nigra* may be important for any practical treatment or prophylactic applications of this research.

Additionally, it should be noted that the attenuated Beaudette strain was used for all experiments presented in this paper. *In vitro* screening using the Beaudette strain has led to the identification of virucidal botanicals that were effective in chicken populations [76]. Therefore, some precedence exists for successful prediction of *in vivo* efficacy using this attenuated strain. This success may be due, in part, to the high amino acid identity (96.3%) between the spike proteins of the Beaudette strain and the highly pathogenic Massachusetts M41 strain of IBV [61]. Experiments using *S. nigra* in chicken populations infected with virulent strains will be important for directly assessing the *in vivo* potential of this plant extract.

Interestingly, while vaccination is the main method for inhibiting IBV in poultry populations [77], its effectiveness on new strains is often minimal, leading to outbreaks in even vaccinated populations [78-80]. Perhaps vaccination in conjunction with administering the active polyphenol could have a synergistic effect, similar to that seen when the polyphenol isoquercetin was administered with the influenza medicine amantadine *in vitro*[24]. Finally, these results have the potential to translate into treatments for other coronaviruses, including those that affect humans. These human coronaviruses (HCoV) include ones that may cause up to 20% of the common cold (HCoV 229E, HCoV OC43); HCoV NL63 and HCoV HKU1, which cause mild to severe respiratory diseases; the SARS CoV, which emerged in 2003 with a 10% mortality rate; and the recently emerged MERS CoV, which currently has a 57% case fatality rate [81,82]. Some evidence supports this idea, in that glycyrrhizin, the active chemical from *G. radix* extracts, inhibited not only IBV, but also the SARS CoV [25,83].

## Conclusions

Taken together, our studies have identified a plant extract from *Sambucus nigra* with previously unknown inhibitory effects against IBV. We have also identified the likely mechanism of this inhibition. Our results could potentially lead to effective treatments or prevention of IBV or similar coronaviruses.

## Abbreviations

IBV: Infectious bronchitis virus; CPE: Cytopathic effect; MOI: Multiplicity of infection; p.i.: Post-infection; pre-C: Treatment of cells with plant extract for 24 h prior to infection; pre-V: Treatment of virus with plant extract for 20 min prior to infection; post: Treatment with plant extract for 24 h following infection.

## Competing interests

No financial or non-financial competing interests exist for any of the authors of this study.

## Authors’ contributions

CC participated in the design of the *S. nigra* portion of the study, carried out most of the *S. nigra* experiments, drafted substantial portions of the manuscript, and helped prepare the final manuscript. DMZ participated in the design of the transmission electron microscope experiments, carried out these experiments, drafted a portion of the manuscript, and helped prepare the final manuscript. SB participated in the design of the *N. sativa* portion of the study, carried out all of the *N. sativa* experiments and some of the *R. rosea* experiments, and helped prepare the final manuscript. MS participated in the design of the *R. rosea* portion of the study, carried out some of the *R. rosea* experiments, and helped prepare the final manuscript. KC drafted a substantial portion of the manuscript and helped prepare the final manuscript. EH participated in the design of the transmission electron microscope experiments and helped prepare the final manuscript. ARP conceived of the study, participated in the design of the study, carried out some of the *S. nigra* experiments, coordinated all authors’ efforts, drafted portions of the manuscript, and helped prepare the final manuscript. All authors read and approved the final manuscript.
